# The Influence of Childhood Aerobic Fitness on Learning and Memory

**DOI:** 10.1371/journal.pone.0072666

**Published:** 2013-09-11

**Authors:** Lauren B. Raine, Hyun Kyu Lee, Brian J. Saliba, Laura Chaddock-Heyman, Charles H. Hillman, Arthur F. Kramer

**Affiliations:** 1 Department of Kinesiology and Community Health, University of Illinois at Urbana - Champaign, Urbana, Illinois, United States of America; 2 The Beckman Institute for Advanced Science and Technology, University of Illinois at Urbana - Champaign, Urbana, Illinois, United States of America; University of São Paulo, Brazil

## Abstract

**Introduction:**

There is a growing trend of inactivity among children, which may not only result in poorer physical health, but also poorer cognitive health. Previous research has shown that lower fitness has been related to decreased cognitive function for tasks requiring perception, memory, and cognitive control as well as lower academic achievement.

**Purpose:**

To investigate the relationship between aerobic fitness, learning, and memory on a task that involved remembering names and locations on a fictitious map. Different learning strategies and recall procedures were employed to better understand fitness effects on learning novel material.

**Methods:**

Forty-eight 9–10 year old children (n = 24 high fit; HF and n = 24 low fit; LF) performed a task requiring them to learn the names of specific regions on a map, under two learning conditions in which they only studied (SO) versus a condition in which they were tested during study (TS). The retention day occurred one day after initial learning and involved two different recall conditions: free recall and cued recall.

**Results:**

There were no differences in performance at initial learning between higher fit and lower fit participants. However, during the retention session higher fit children outperformed lower fit children, particularly when the initial learning strategy involved relatively poor recall performance (i.e., study only versus test-study strategy).

**Conclusions:**

We interpret these novel data to suggest that fitness can boost learning and memory of children and that these fitness-associated performance benefits are largest in conditions in which initial learning is the most challenging. Such data have important implications for both educational practice and policy.

## Introduction

There is a growing trend of inactivity among children, leading to an increased likelihood of being overweight and unfit [1], [2], [3], [4]. Such inactivity not only results in poor physical health, but may also result in poor cognitive health, with an emerging literature suggesting a link between these aspects of health (see [5] for review). In children, lower amounts of fitness have been related to decreased cognitive function for tasks requiring perception, memory, and cognitive control [6], [7] as well as lower academic achievement [8], [9], [10]. However, our understanding of which aspects of cognition are modifiable through fitness is in its infancy, as only a few aspects of memory have been studied.

Memory is a broad term that encompasses a varied set of processes, which reflect the capacity of an organism to benefit and learn from their past experiences, and consists of procedural, semantic, and episodic systems [11]. These processes are supported by unique, but corresponding neuronal networks [12], which include the hippocampus. The hippocampus is important for learning [13], [14], the retrieval of deeply encoded items [15], episodic memory [16], and novelty detection [17], [18].

Furthermore, memory processes are comprised of encoding, storage, and recall. While encoding and recall are active processes that occur at a specific point in time [19], storage is a temporally distributed transient process [20]. Encoding involves the processing of information while recall requires access to the information that has been encoded into memory [21].

An individual’s performance on tasks that tap some aspects of memory, as well as the size of their hippocampus, is to some extent modifiable [22], [23], [24]. One factor that has been shown to modify memory performance is exercise, which has been found to impact memory function across the lifespan in both humans and rodents [22], [23], [25]. Exercising mice have been found to have enhanced spatial learning and memory, including better performance on the Morris water maze task, and increased neurogenesis (which may contribute to enhanced learning) [26], [27], [28], [29], [30]. Wheel running in mice has further been shown to increase hippocampal levels of insulin like growth factor (IGF), brain derived neurotrophic factors (BDNF); molecules involved in angiogenesis, neuronal survival, synaptic development, learning and memory [31], [32], [30], [33]. The benefits of exercise on memory extend to children as well. During an encoding task, higher fit children utilized more successful encoding and recall strategies, suggesting that higher fit children may have stronger cognitive control abilities and may use memory more efficiently [22].

A method to enhance encoding is testing, as the retrieval processes during testing may benefit memory more than additional study time. This method is known as the ‘testing effect’ [34], [35], [36], [37]. The process of testing during encoding effectively enhances memory and is critical for long term memory, as repeated studying does not enhance long term memory to the same degree [38]. Carpenter and Pashler [39] employed testing during a visuo-spatial task and demonstrated that testing during the encoding phase produced significant enhancements in learning. The testing effect has been extended to children, such that material encoded using intermittent recall tests was remembered better than material that had been encoded using repeated study [40].

Interestingly, while fitness has been shown to be positively related to a number of aspects of cognition [5], [6], [8], [9], [22], [31], the influence of fitness on learning has not been explicitly examined in human studies. Therefore, it is an open question as to whether fitness engenders more effective learning. To address this question we examined learning in a study-only and test-study memory paradigm with children who differed in fitness level.

Children were asked to learn novel names of regions and locations on a fictitious map. The children initially learned this information either by studying the maps or by interleaved studying and testing of the map regions and locations. The children’s memory for this information was tested with both free recall and cued recall procedures.

We hypothesized that fitness would enhance learning and memory of children, particularly in the most challenging situations. The results should also be informative with regard to whether fitness-based learning enhancement occurs during initial learning, at retention or at both points of time. Finally, the present study provides an initial examination of the potential interaction between fitness and learning strategies that have been found to have a differential influence on learning success.

## Methods

### Participants

Forty-nine children (27 females) ages 9–10 years were recruited for this study. The study described herin was conducted with prior approval of the University of Illinois at Urbana- Champaign Human Subjects Committee. All participants and their legal guardians completed written assent and consent prior to participation in the study in accordance with the Institutional Review Board at the University of Illinois Urbana-Champaign and their rights were protected. Learning and recall sessions (days 2 and 3, respectively) occurred on consecutive days during which participants were asked to refrain from any structured physical activity, as acute aerobic exercise has previously been found to influence cognition [41]. Fitness and demographic information for all participants may be found in Table 1. Participants qualified for the study if they were either higher (n = 24; HF) or lower (n = 24; LF) fit, which was determined using a maximal oxygen consumption test completed on a motorized treadmill. Fitness data were evaluated relative to age and gender normative values provided by Shvartz and Reibold [42]. HF participants were classified as those scoring above the 70^th^ percentile for their age and gender, while LF participants were those scoring below the 30^th^ percentile [42]. Participants falling between the 30^th^ and 70^th^ percentile were not included in the study.

**Table 1:** Mean (SE) Demographic Information for Higher Fit (HF) and Lower Fit (LF) Groups.

Measure	HF	LF
*n*	24 (14 female)	24 (12 female)
Age (years)	9.9 (0.1)	9.9 (0.6)
Socioeconomic Status (SES)	2.4 (0.1)	2.1 (0.1)
KBIT (IQ)	120.7 (2.2)	116.4 (1.4)
ADHD	35.0 (5.9)	33.3 (4.1)
VO_2_ max Relative*	51.4 (1.0)	37.3 (0.9)
VO_2_ max Percentile*	82.0 (1.3)	10.5 (1.4)

* Indicates significant difference, p<0.05.

### Aerobic Fitness

Participants completed a VO_2_max test using an indirect calorimetry system (Parvo Medics True Max 2400, Sandy, UT) on a motorized treadmill. The test was administered using a modified Balke protocol [43] and participants ran at a constant speed with incremental grade inclines of 2.5% every two minutes until volitional fatigue. Average oxygen uptake (VO_2_) and respiratory exchange ratio (RER) were assessed every 20 seconds, and participants wore a polar heart rate (HR) monitor (Model A1, Polar Electro, Finland) throughout the test. Every two minutes, ratings of perceived exertion were taken based on the children’s OMNI scale [44], which ranges from one to ten, with one being “a little tired” and ten being “very very tired” along with pictographs to represent perceived physical effort. Participants were instructed to point to the picture that indicated how tired they felt. Relative peak oxygen consumption was expressed in ml/kg/min. Relative VO_2_max was evidenced by achieving two of the following four criteria: a) a plateau in oxygen consumption corresponding to an increase of less than 2 ml/kg/min despite an increase in workload; b) RER≥1.0 [45]; c) a peak HR≥185 beats per minute (bpm) [43] and a HR plateau [46]; and/or d) RPE≥8 [44].

### Map Task

Learning and recall sessions occurred on consecutive days and involved the mobile application use of an iPad (Apple Inc., Cupertino, CA). The task involved remembering names of specific regions, which were comprised of four letters each (e.g., Kent), from a map. Within each map, a total of 10 regions were named from the overall 20 region map. All testing materials were fictional, and a total of four maps were used (two for practice). Learning condition refers to the strategy with which the map was learned, either the study only (SO) strategy or the test study (TS) strategy. Recall type refers to the manner in which the map was recalled: free recall or cued recall. A within-subjects design was utilized such that each child learned one map using the SO strategy and one map using the TS strategy, and recalled each map using both free and cued recall. The order in which the maps were learned was counterbalanced across HF and LF participants.

### Procedure

#### Day 1

Following consent, the participants’ legal guardians completed questionnaires including: the Pre Participation Health Screening [47], the Attention Deficit Hyperactive Disorder Rating Scale IV (ADHD Rating Scale IV; [48]), and a health history and demographics questionnaire. Participants’ socio-economic status (SES) was determined using a trichotomous index based on 1) participation in free or reduced price lunch program at school, 2) the highest level of education obtained by the mother and father, and 3) the number of parents who work full time [49]. Socio-economic status was confirmed by a second measure, which provided total household income. Together, legal guardians and participants completed the Modified Tanner Staging System [50] to indicate that the participants’ pubertal status was at or below a score of 2 at the time of testing (i.e., prepubescent). Participants were administered the Kaufman Brief Intelligence Test-II [51] to measure general intelligence and screen for children who may have a lower than normal intelligence score or a learning disability. Lastly, participants completed a maximal oxygen consumption (VO2max) test to assess their level of aerobic fitness.

#### Day 2: Learning Day

The second day of the experiment required learning the names and locations of the regions. Participants learned two different maps using two different learning strategies: a study only (SO) strategy and a test study (TS) strategy [40]. Each learning strategy began with an explanation and practice on a separate sample map. During the practice block, the task was described and participants were allowed to become familiar with the learning protocol. Participants were allowed to ask questions and were monitored to ensure they understood the directions. During each learning strategy, participants were asked to attend to the map and remember the regions for subsequent learning blocks. The two different maps were counterbalanced between the SO and TS strategies, along with the SO and TS strategy order, such that each participant was randomly assigned to one of four different learning orders. Counterbalancing occurred within each fitness group to insure that the orders were equal across groups.

The SO learning strategy began with an initial exposure of the map in which participants observed each region and name individually for three seconds, and were instructed to try and remember everything about the map as best they could. Following initial exposure, the SO learning condition began. In the SO condition, participants cycled through ten regions on the map for six blocks. Importantly, the SO learning condition consisted of only map study, the region name was displayed in its correct location on the map for 6 seconds and participants were instructed to tap on the region. The tap was utilized during this condition to ensure that participants were attending to the map and paying attention to the task. A timer was presented on the screen to make participants aware of how much time remained. Participants were instructed to try and remember where the name was for subsequent blocks and for the following day. This process was repeated for each of the ten regions for six blocks.

The TS learning strategy began with an initial exposure of the map in which participants observed each region and name individually for three seconds, and were instructed to try and remember everything about the map as best they could. Following initial exposure, the TS learning strategy began. In the TS condition, participants cycled through ten regions on the map for six blocks. During each block, region names were displayed for 6 seconds each, which included a 4 second test phase and a 2 second study phase. During the 4 second test phase, the region name was displayed on the screen outside of the map regions. Participants were instructed to tap in the region on the map that correctly identified the name and this response was recorded. Participants had 4 seconds to respond, and they were aware of how much time remained through the inclusion of a timer beside the region name. Following the test phase, the region name was shown in its correct region location on the map for 2 seconds during the study phase. Again, participants were told to try and remember where the name was for subsequent blocks and for the following day. This process was repeated for each of the ten regions for six blocks. The unique aspect of the TS strategy is that it involves tests to assess how much the child is remembering. Performance was measured by the number of correctly identified regions during each block.

Both the SO and the TS conditions required the same amount of time and emphasized each region name for the same amount of time. In both conditions, the region names were presented so that their appearance varied in that no two regions appeared consecutively on concurrent blocks (i.e., the last region name of one block was different from the first region name of the next block) and the same region name only appeared once per block. The map and region outlines remained on the screen for the entire condition. However, the learning strategies differed in that the SO strategy simply provided additional study opportunities whereas the TS strategy included tests interspersed with study opportunities.

#### Day 3: Recall Day

Participants returned one day after studying the maps to complete the recall condition. There were two types of recall tests administered for each map, a free recall test and a cued recall test. Free recall tests, thought to require more transfer (since free recall was not used during initial learning of the map regions), were administered first, followed by cued recall tests, which require little transfer. The order in which the maps were tested was counterbalanced such that half of the participants were first tested on the map learned in the SO condition and other half of the participants were first tested on the map learned in the TS condition.

During free recall, participants were shown an unlabeled, outlined map on the iPad. Text boxes were displayed in the regions and participants were instructed to type the region name as accurately as they could. Participants clicked in each text box and typed the region names from memory.

In the cued recall condition, the participants viewed the same unlabeled maps with text boxes, however the ten region names for each map were presented below each map to serve as a word bank for the participants. Participants were instructed to type the region names in the appropriate text box as accurately as they could. Participants clicked in each text box and typed the region names with available assistance from the word bank.

### Statistical Analysis

A general linear mixed model (GLM) approach with a repeated measures design was used in SPSS v.19 (Chicago, IL). Accuracy measures were entered as repeated measures using a 2 (learning: SO, TS) x 2 (recall: free, cued) factorial model with fitness (HF, LF) entered as the between subjects factor. Findings with three or more within-subject levels are reported using the Greenhouse-Geisser correction statistic for violations of sphericity. Response accuracy for the TS condition during the learning day was assessed using a 2 (Fitness: HF, LF) x 6 (Study Block: 1–6) repeated measures ANOVA for the test study condition. Accuracy on the recall day was assessed using a 2 (Fitness: HF, LF) x 2 (Recall Type: Free, Cued) x 2 (Learning Condition: TS, SO) repeated measures ANOVA. Family-wise alpha levels were set at p = 0.05 and post hoc comparisons were conducted using Bonferroni correction.

## Results

### Day1: Demographic Information

Fitness and demographic information can be found in Table 1. Significant differences were not observed for demographic variables between the HF and LF groups. Specifically, HF and LF children did not differ in terms of age, gender, SES, ADHD scores, pubertal timing, or KBIT II (all p>0.05). One child (LF) was excluded from analysis due to a KBIT II score greater than 3 standard deviations below the mean. Confirming group selection, HF and LF groups differed significantly in their VO_2_max percentile, with HF children having a greater VO_2_max percentile (82.0%±1.3%) than LF children (10.5%±1.4%).

### Day2: Learning Session

**Figure 1:**
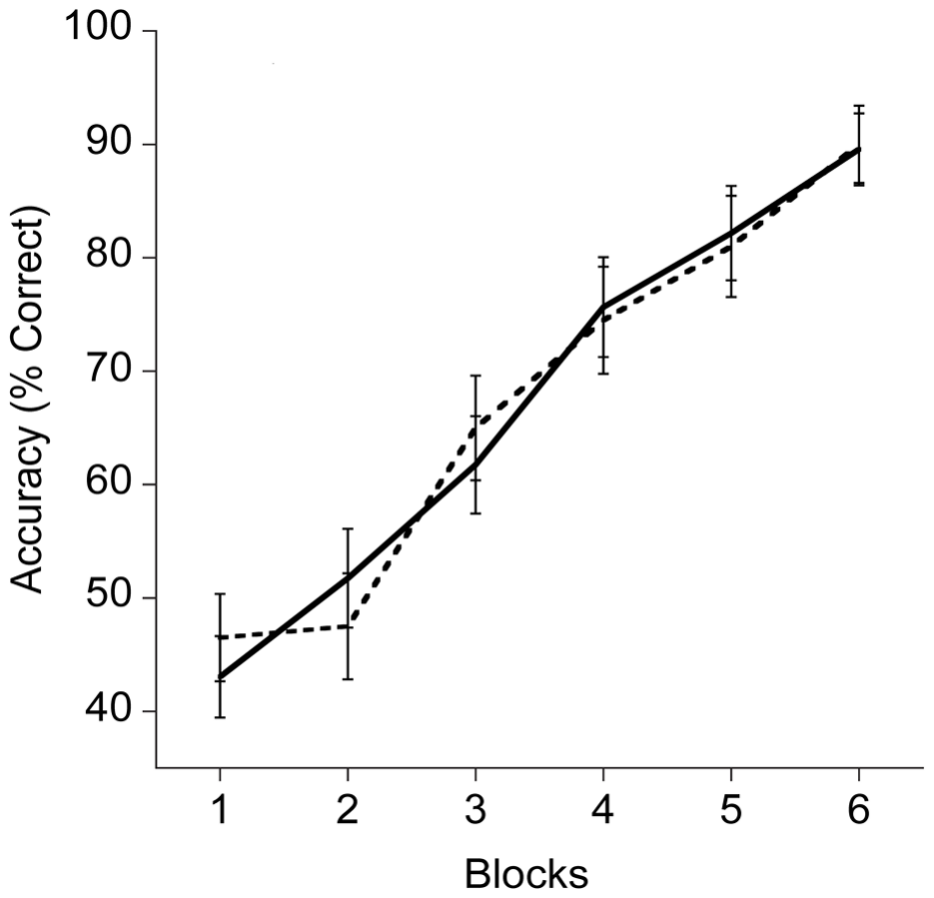
Response accuracy for higher fit and lower fit groups using the test-study encoding strategy. Higher (HF) fit performance is shown in solid lines, lower fit (LF) is shown in dashed lines.

The omnibus analysis revealed a main effect of Study Block in the TS learning condition, F(4.1, 174)  = 107.3, p≤0.005, η^2^ = 0.719. Post hoc analysis indicated that all means were significantly different, t’s(43) ≥1.9, p≤0.005, such that performance improved with subsequent blocks during the TS learning condition (see Figure 1).

### Day 3: Recall Session

The ANOVA revealed three main effects: Fitness, F(1, 46)  = 3.9, p = 0.05, η^2^ = 0.08, with HF participants (54.2%±3.6%) having greater accuracy than LF participants (44.2%±3.6%); Learning Condition, F(1, 46)  = 89.5, p≤0.005, η^2^ = .66, with TS learning condition (64.0%±2.6%) resulting in higher accuracy than SO learning condition (34.4%±3.3%); and Recall Type, F(1, 46)  = 328.1, p≤0.005, η^2^ = 0.88, with cued recall (67.6%±2.8%) having higher accuracy than free recall (30.7%±2.7%). In addition, there was an interaction of Fitness x initial Learning Condition, F(1, 46)  = 5.2, p = 0.03, η^2^ = 0.10. Decomposition of this interaction revealed that HF children performed more accurately (43.0%±5.5%) than LF children (25.8%±3.6%) during recall of the map that was learned using the SO strategy [t’s(46)>2.6, p = 0.012]. This effect remained significant following Bonferroni correction. No accuracy differences were observed between groups during recall of the condition learned using the TS strategy [t’s(46) >0.56, p = 0.57].

Further, an interaction of Learning Condition x Recall Type was observed, F(1, 46)  = 8.0, p = 0.01, η^2^ = 0.15. Post hoc analysis indicated the map learned in the SO condition and tested with free recall (18.8%±3.4%) had decreased accuracy relative to the map learned in the TS condition and tested with free recall (42.7%±3.2%), t’s(47)>6.3, p<0.0083; decreased accuracy relative to the map learned in the SO condition and tested with cued recall (50.1%±4.0%), t’s(47)>12.9, p<0.0083; and decreased accuracy relative to the map learned in the TS condition and tested with cued recall (85.2%±2.8%), t’s(47)>17.4, p<0.0083. Accuracy for the map learned in the TS condition and tested with free recall (42.1%±3.2%) did not differ from accuracy for the map learned in the SO condition and tested with cued recall (50.1%±4.0%), t’s(47)>1.9, p>0.0083, but did show decreased accuracy relative to the map learned in the TS condition and tested with cued recall (85.2%±2.8%), t’s(47)>13.5, p<0.0083. Accuracy for the map learned in the SO condition and tested with cued recall (50.1% ±4.0%) showed decreased accuracy relative to the map learned in the TS condition and tested with cued recall (85.2%±2.8%), t’s(47)>9.2, p<0.0083.

## Discussion

The current findings indicate that interspersed testing and study as well as higher levels of aerobic fitness benefits learning and memory. Consistent with the extant literature [38], [39], [40], participants performed best on later recall of novel visuo-spatial material using an initial learning strategy that included both testing and study periods. Indeed, the current results replicate those of Rohrer [39] and suggest that in children, testing during encoding improves later recall ability. Also consistent with the literature, cueing enhanced recall for both of the study conditions.

**Figure 2:**
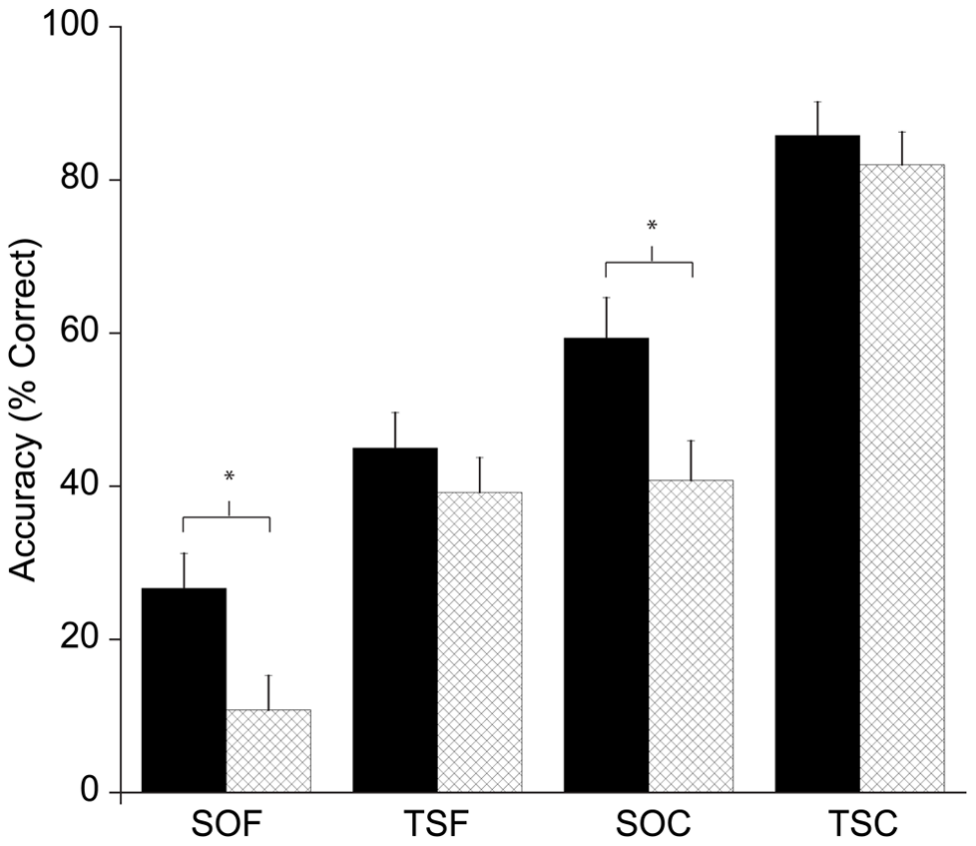
Response accuracy for higher fit and lower fit groups on recall day by encoding strategy. Higher fit performance is represented by the black bars, and lower fit performance is represented by the light bars. SOF represents the map learned using the study only strategy and tested with free recall; TSF represents the map learned using the test study strategy and tested with free recall; SOC represents the map learned using the study only strategy and tested using cued recall; TSC represents the map learned in the test study strategy and tested using cued recall.

**Figure 3:**
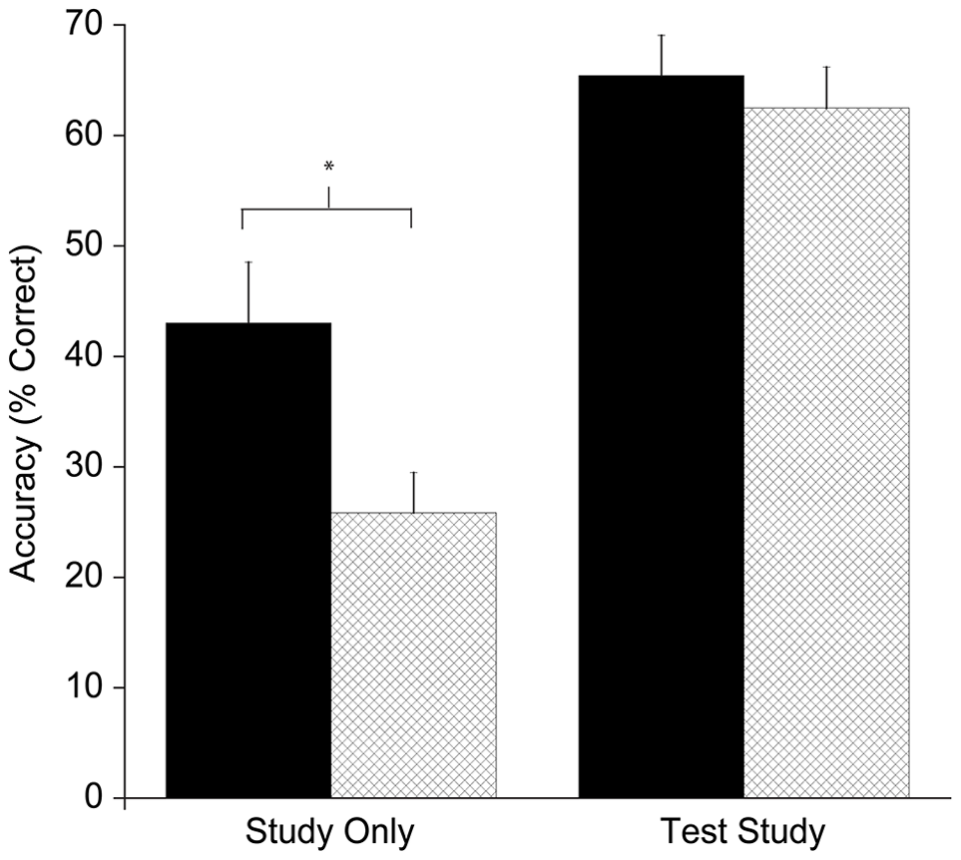
Recall accuracy for higher fit and lower fit groups on the map learned using the study-only strategy. Higher fit performance is represented by the black bars, and lower fit performance is represented by the light bars.

The main focus of the present study was the influence of fitness differences on learning and memory, both during the initial encoding phase with the map task and with delayed recall on the day following initial learning. As indicated in Figure 1, fitness differences were not observed in the first session in which children were exposed to the map learning. Such data suggest that initial encoding and recall may not be influenced by fitness level, providing important boundary conditions on fitness effects on cognition. However, as indicated in Figures 2 and 3, fitness differences were clearly revealed during delayed recall (on day three of the study).

Interestingly, fitness differences interacted with initial learning strategy, with higher fit children outperforming lower fit children in recall of the regions learned using the study only condition, while higher and lower fit children performed similarly in recall of the regions learned using the test-study condition. These data might be interpreted to suggest that higher levels of fitness have their greatest impact in the most challenging situations; in the present case when a relatively ineffective initial learning strategy was employed (i.e., the study only as compared to the test-study condition). Additionally, participants performed best when recall was explicitly cued with the list of region names (as compared to the free recall condition; see Figure 2). However, fitness did not interact with the cue condition. Therefore, it would appear that fitness does not assist children with learning in all challenging situations. Indeed, when both the initial learning conditions and cueing effects are considered together, it would appear that fitness might primarily exert its beneficial influence at initial encoding of novel material to a greater extent than during retrieval (i.e., the cueing conditions).

This conclusion is consistent with both the animal [26], [27], [29], [30] and human [22], [24], [23], [25] studies, which suggest that fitness and exercise has a significant influence on hippocampal structure and function. Hippocampus is responsible, in part, for encoding information into memory, and in particular for relations among different aspects of the environment (e.g., such as a face, with a name, with a profession [13], [14]). Indeed, the encoding and representation of region names with locations in the present study is clearly the kind of information that has been shown to be well served by a highly functioning hippocampus.

In sum, the findings described herein suggest two important factors, initial learning strategy and fitness, for improving learning in children who may struggle with certain subject matters or require additional educational assistance. Future research should focus on the manner in which these factors impact the neural processes of children during learning and how differential neural networks associated with learning strategy, fitness, and their interaction impact both short and long term retention as well as the transfer of learned information and skills beyond the initial learning situation. The findings in the present study are also important from an educational policy perspective. Reducing or eliminating physical education in schools, as is often done in tight financial times, may not be the best way to ensure educational success among our young people.

## References

[1] Department of Health and Human Services [DHHS] and Department of Education [DOE] (2000) Promoting better health for young people through physical activity and sports. A Report to the President from the Secretary of Health and Human Services and the Secretary of Education. Silver Spring, MD: Centers for Disease Control.

[2] Olshansky SJ, Passaro DJ, Hershow RC, Layden J, Carnes BA, et al (2005) A potential decline in life expectancy in the United States in the 21st century. N Engl J Med 352: 1138–1145. 15784668 10.1056/NEJMsr043743

[3] Baker JL, Olsen LW, Sorensen TIA (2007) Childhood body- mass index and the risk of coronary heart disease in adulthood. N Engl J Med 357: 2329–2337. 18057335 10.1056/NEJMoa072515 PMC3062903

[4] Ludwig DS (2007) Childhood obesity- the shape of things to come. N Engl J Med 357: 2325–2327. 18057334 10.1056/NEJMp0706538

[5] Hillman CH, Erickson KI, Kramer AF (2008) Be smart, exercise your heart: exercise effects on brain and cognition. Nat Rev Neurosci 9: 58–65. 18094706 10.1038/nrn2298

[6] Hillman CH, Buck SM, Themanson JR, Pontifex MB, Castelli DM (2009) Aerobic fitness and cognitive development: Event-related brain potential and task performance indices of executive control in preadolescent children. Dev Psychol 45: 114–129. 19209995 10.1037/a0014437

[7] Kamijo K, Pontifex MB, Khan NA, Raine LB, Scudder MR, et al.. (2012) The association of childhood obesity to neuroelectric indices of inhibition. Psychophysiology. 10.1111/j.1469-8986.2012.01459.x 22913478

[8] Castelli DM, Hillman CH, Buck SM, Erwin HE (2007) Physical fitness and academic achievement in third- and fifth-grade students. J Sport Exerc Psychol 29: 239–252. 17568069 10.1123/jsep.29.2.239

[9] Sibley BA, Etnier JL (2003) The relationship between physical activity and cognition in children: A meta-analysis. Pediatr Exerc Sci 15: 243–256.

[10] Kamijo K, Khan NA, Pontifex MB, Scudder MR, Drollette ES, et al.. (2012) The relation of adiposity to cognitive control and scholastic achievement in preadolescent children. Obesity. 10.1038/oby.2012.112 PMC3414677 22546743

[11] Tulving E (1985) How many memory systems are there? Am Psychol 40: 385–398.

[12] Henke K, Buck A, Weber B, Wieser HG (1997) Human hippocampus establishes associations in memory. Hippocampus 7: 249–256. 9228523 10.1002/(SICI)1098-1063(1997)7:3<249::AID-HIPO1>3.0.CO;2-G

[13] Cohen NJ, Eichenbaum H (1993) Memory, Amnesia, and the Hippocampal System. MIT Press, Cambridge.

[14] Jarrard LE (1995) What does the hippocampus really do? Behav Brain Res 71: 1–10. 8747170 10.1016/0166-4328(95)00034-8

[15] Rugg MD, Fletch PC, Frith CD, Frackowiak RSJ, Dolan RJ (1996) Differential activation of the prefrontal cortex in successful and unsuccessful memory retrieval. Brain 119: 2073–2083. 9010011 10.1093/brain/119.6.2073

[16] Vargha-Khadem F, Gadian DG, Watkins KE, Connelly A, Van Paesschen W, et al (1997) Differential effects of early hippocampal pathology on episodic and semantic memory. Science 277: 376–380. 9219696 10.1126/science.277.5324.376

[17] Tulving E, Markowitz HJ, Craik FIM, Habib R, Houlse S (1996) Novelty and familiarity activations in PET studies of memory encoding and retrieval. Cereb Cortex 6: 71–79. 8670640 10.1093/cercor/6.1.71

[18] Grunwald T, Lehnertz K, Heinze HJ, Helmstaedter C, Elger CE (1998) Verbal novelty detection within the human hippocampus proper. Proc Natl Acad Sci USA 95: 3193. 9501239 10.1073/pnas.95.6.3193 PMC19718

[19] Tulving E (1983) Elements of episodic memory. Oxford: Clarendon Press. 56–62.

[20] Gluck MA, Myers CE (1997) Psychobiological models of hippocampal function in learning and memory. Annu Rev Psychol 48: 481. 9046567 10.1146/annurev.psych.48.1.481

[21] Polster MR, Nadel L, Schacter DL (1991) Cognitive neuroscience analyses of memory: A historical perspective. J Cogn Neurosci 3: 95–116. 23972087 10.1162/jocn.1991.3.2.95

[22] Chaddock L, Erickson KI, Prakash RS, Kim JS, Voss MW, et al (2010) A neuroimaging investigation of the association between aerobic fitness, hippocampal volume, and memory performance in preadolescent children. Brain Res 1358: 172–183. 20735996 10.1016/j.brainres.2010.08.049 PMC3953557

[23] Erickson KI, Prakash RS, Voss MW, Chaddock L, Hu L, et al (2009) Aerobic fitness is associated with hippocampal volume in elderly humans. Hippocampus 19: 1–10. 19123237 10.1002/hipo.20547 PMC3072565

[24] Erickson KI, Voss MW, Prakash RS, Basak C, Szabo A, et al (2011) Exercise training increases size of hippocampus and improves memory. Proc Natl Acad Sci U S A 108: 3017–3022. 21282661 10.1073/pnas.1015950108 PMC3041121

[25] Pereira AC, Huddleston DE, Brickman AM, Sosunov AA, Hen R, et al (2007) An in vivo correlate of exercise-induced neurogenesis in the adult dentate gyrus. Proc Natl Acad Sci U S A 104: 5638–5643. 17374720 10.1073/pnas.0611721104 PMC1838482

[26] Fordyce DE, Farrar RP (1991) Physical activity effects on hippocampal and parietal cortical cholinergic function and spatial learning in F344 rats. Behav Brain Res 43: 115–123. 1867753 10.1016/s0166-4328(05)80061-0

[27] Fordyce DE, Wehner JM (1993) Physical activity enhances spatial learning performance with an associated alteration in hippocampal protein kinase C activity in C57BL/6 and DBA/2 mice. Brain Res 619: 111–119. 8374769 10.1016/0006-8993(93)91602-o

[28] Van Praag H, Kempermann G, Gage FH (1999) Running increases cell proliferation and neurogenesis in the adult mouse dentate gyrus. Nat Neurosci 2: 266–270. 10195220 10.1038/6368

[29] Van Praag H, Shubert T, Zhao C, Gage FH (2005) Exercise enhances learning and hippocampal neurogenesis in aged mice. J Neurosci 25: 8680–8685. 16177036 10.1523/JNEUROSCI.1731-05.2005 PMC1360197

[30] Vaynman S, Ying Z, Gomez-Pinilla F (2004) Hippocampal BDNF mediates the efficacy of exercise on synaptic plasticity and cognition. Eur J Neurosci 20: 2580–2590. 15548201 10.1111/j.1460-9568.2004.03720.x

[31] Cotman CW, Berchtold NC (2002) Exercise: a behavioral intervention to enhance brain health and plasticity. Trends Neurosci 25: 295–301. 12086747 10.1016/s0166-2236(02)02143-4

[32] Neeper SA, Gomez-Pinilla F, Choi J, Cotman C (1995) Exercise and brain neurotrophins. Nature 373: 109. 7816089 10.1038/373109a0

[33] Berchtold NC, Chinn G, Chou M, Kesslak JP, Cotman CW (2005) Exercise primes a molecular memory for brain-derived neurotrophic factor protein induction in the rat hippocampus. Neuroscience 133: 853–861. 15896913 10.1016/j.neuroscience.2005.03.026

[34] Carpenter SK, DeLosh EL (2006) Impoverished cue support enhances subsequent retention: Support for the elaborative retrieval explanation of the testing effect. Mem Cognit 34: 268–276. 10.3758/bf03193405 16752591

[35] Roediger HL, Karpicke JD (2006) The power of testing memory: Basic research and implications for educational practice. Perspect Psychol Sci 1: 181–210. 26151629 10.1111/j.1745-6916.2006.00012.x

[36] Carrier M, Pashler H (1992) The influence of retrieval on retention. Mem Cognit 20: 632–642. 10.3758/bf03202713 1435266

[37] McDaniel MA, Masson MEJ (1985) Altering memory representations through retrieval. J Exp Psychol Learn Mem Cogn 11: 371–385.

[38] Karpicke JD, Blunt JR (2011) Retrieval practice produces more learning than elaborative studying with concept mapping. Science 331: 772–775. 21252317 10.1126/science.1199327

[39] Carpenter SK, Pashler H (2007) Testing beyond words: Using tests to enhance visuospatial map learning. Psychon Bull Rev 14: 474–478. 17874591 10.3758/bf03194092

[40] Rohrer D, Taylor K, Sholar B (2010) Tests enhance the transfer of learning. J Exp Psychol Learn Mem Cogn 36: 233. 20053059 10.1037/a0017678

[41] Hillman CH, Pontifex MB, Raine LB, Castelli DM, Hall EE, et al (2009) The effect of acute treadmill walking on cognitive control and academic achievement in preadolescent children. Neuroscience 159: 1044. 19356688 10.1016/j.neuroscience.2009.01.057 PMC2667807

[42] Shvartz E, Reibold RC (1990) Aerobic fitness norms for males and females aged 6 to 75 years: A review. Aviat Space Environ Med 61: 3–11. 2405832

[43] American College of Sports Medicine (2010) ACSM’s guidelines for exercise testing and prescription (8th ed.). New York: Lippincott Williams & Wilkins.

[44] Utter AC, Robertson RJ, Nieman DC, Kang J (2002) Children's OMNI scale of perceived exertion: walking/running evaluation. Med Sci Sports Exerc 34: 139–144. 11782659 10.1097/00005768-200201000-00021

[45] Bar-Or O (1983) Pediatric sports medicine for the practitioner: From physiologic principles to clinical applications. New York: Springer-Verlag.

[46] Freedson PS, Goodman TL (1993) Measurement of oxygen consumption. In: Rowland TW, editor. Pediatric laboratory exercise testing: Clincal Guidelines. Champaign, Il: Human Kinestics. 91–113.

[47] Healthy Active Living and Obesity (HALO) Research Group (2010) Pre-Participation Health Screening for Children. Children’s Hospital of Eastern Ontario Research Institute.

[48] DuPaul GJ, Power TJ, Anastopoulos AD, Reid R (1998) ADHD Rating Scale-IV: Checklists, norms, and clinical interpretation. New York: Guilford Press.

[49] Birnbaum AS, Lytle LA, Murray DM, Story M, Perry CL, et al (2002) Survey development for assessing correlates of young adolescents' eating. Am J Health Behav 26: 284–295. 12081361 10.5993/ajhb.26.4.5

[50] Taylor SJ, Whincup PH, Hindmarsh PC, Lampe F, Odoki K, et al (2001) Performance of a new pubertal self-assessment questionnaire: a preliminary study. Paediatr Perinat Epidemiol 15: 88–94. 11237120 10.1046/j.1365-3016.2001.00317.x

[51] Kaufman AS, Kaufman NL (2004) Kaufman Brief Intelligence Test Second Edition Manual. Bloomington, MN: NCS Pearson.

